# LINC01929 Is a Prognostic Biomarker for Multiple Tumours and Promotes Cell Proliferation in Breast Cancer Through the TNF/STAT3 Axis

**DOI:** 10.1111/jcmm.70227

**Published:** 2024-11-25

**Authors:** Yanlin Gu, Zhengyang Feng, Xiaoyan Xu, Liyan Jin, Guoqin Jiang

**Affiliations:** ^1^ Department of Thyroid and Breast Surgery The Second Affiliated Hospital of Soochow University Suzhou China; ^2^ Department of Oncology The Second Affiliated Hospital of Soochow University Suzhou China; ^3^ Department of Operating Room Traditional Chinese Medicine Hospital of Kunshan Kunshan China

**Keywords:** biomarker, cancer, prognosis

## Abstract

The aim of this study was to investigate whether long intergenic non‐coding RNA 1929 (LINC01929), a novel long non‐coding RNA, could serve as a prognostic biomarker for various tumours and explore its function. The expression and prognosis of LINC01929 across 33 different tumour types in patients in the Cancer Genome Atlas (TCGA) database were analysed. Also, the correlation between LINC01929 expression, tumour mutational burden (TMB), microsatellite instability (MSI), immune checkpoint status and immune cell infiltration was examined. Moreover, the function of LINC01929 in the breast cancer cell lines was explored via CCK‐8, colony formation and cell cycle assays. In addition, the downstream mechanisms of LINC01929 were analysed via transcriptome sequencing, RT–qPCR, and western blotting. Our analysis revealed that LINC01929 was weakly expressed in 3 tumour types and highly expressed in 14 tumour types, and low expression of LINC01929 was correlated with better clinical outcomes in 15 tumour types. Furthermore, LINC01929 expression was correlated significantly with the TMB, MSI, immune checkpoint and immune cell infiltration across multiple tumour types. The knockdown of LINC01929 inhibited cell cycle progression, cell proliferation, and tumorigenesis and downregulated the TNF pathway and STAT3 expression. The treatment with exogenous TNF‐α partially reversed the cell cycle progression and proliferation inhibition caused by LINC01929 knockdown, and these effects were accompanied by changes in STAT3 expression. LINC01929 may serve as an effective biomarker affecting the TMB, MSI, immune cell infiltration and immune checkpoint status. Mechanistically, LINC01929 affects cell cycle progression and cell proliferation through the TNF/STAT3 axis. These findings offer valuable insights into the potential applications of LINC01929 in tumour therapy, which may yield novel targets and strategies for the diagnosis and treatment of patients.

## 1 Introduction

The incidence and progression of cancer, a profoundly complex disease, are impacted by various factors, and cancer remains a leading cause of morbidity and mortality worldwide, thus exerting a substantial burden on public health [1]. The high prevalence and lethality of cancer underscore the urgent need for identifying novel biomarkers and therapeutic targets, aiming to improve patients' outcomes. Despite advancements in cancer treatment, the prognosis for many cancer types remains poor, particularly in advanced stages [2]. Although different types of cancer exhibit different pathological characteristics, accumulating evidence suggests that commonalities and interdependencies exist across cancer types [3]. Pancancer research aims to synthesise commonalities, analyse individualised features and explore shared mechanisms underlying the aetiology and progression of various cancer types. Consequently, this approach offers invaluable support for shared cancer treatment and precision medicine [4, 5].

In recent years, the realm of tumour research has witnessed remarkable advancements in the identification and application of biomarkers. Notably, circulating tumour DNA (ctDNA) has emerged as a pivotal tool for the early detection and continuous monitoring of the dynamic fluctuations within tumours [6, 7, 8]. Concurrently, the evaluation of tumour mutation burden (TMB) and microsatellite instability (MSI) has provided critical insights that inform the selection of strategies [9, 10]. In addition, the swift progression of bioinformatics has facilitated the efficient identification of potential markers for tumour diagnosis and treatment, harmonising high‐throughput sequencing with clinical data [11, 12]. These groundbreaking developments herald new avenues for enhancing the diagnosis and treatment of cancer. Long intergenic non‐coding RNA 1929 (LINC01929) is a long non‐coding RNA (lncRNA), and it is longer than 200 nucleotides in length and does not encode proteins but is involved in regulating gene expression at various levels [13]. In recent years, LINC01929 has garnered attention for its potential role in cancer biology [14, 15, 16, 17, 18]. Studies have indicated that LINC01929 is upregulated in several cancer types, including non‐small cell lung carcinoma, oral squamous cell carcinoma, and bladder cancer [15, 17, 18].

Motivated by these findings, this study aimed to assess the influence of LINC01929 on the prognosis across different cancer types and its association with TMB, MSI, immune checkpoints and infiltrated immune cells. Our comprehensive analysis indicated that LINC01929 is highly expressed across various tumour types and may serve as an oncogenic biomarker impacting patient outcomes. Additionally, the intricate relationships between LINC01929 and immune checkpoint molecule expression, TMB, and MSI were explored. To gain further insights into the mechanisms by which LINC01929 promotes cancer progression, transcriptomic sequencing and experimental validations were performed using breast cancer cell lines. The use of multiple analytical methods provides a robust framework for understanding the multifaceted role of LINC01929 in cancer. The integration of in vitro and in vivo experiments further strengthened the study by offering mechanistic insights and validating bioinformatics findings. The ultimate goal of this research is to identify LINC01929 as a potential biomarker for cancer prognosis and a therapeutic target, thereby contributing to the development of more effective cancer treatments.

## 2 Materials and Methods

### 2.1 Expression Analysis of LINC01929

The RNA‐seq data of 33 various cancer (adrenocortical carcinoma (ACC), bladder urothelial carcinoma (BLCA), breast invasive carcinoma (BRCA), cervical squamous cell carcinoma and endocervical adenocarcinoma (CESC), cholangiocarcinoma (CHOL), colon adenocarcinoma (COAD), diffuse large B‐cell lymphoma (DLBC), oesophageal carcinoma (ESCA), glioblastoma multiforme (GBM), head and neck squamous cell carcinoma (HNSC), kidney chromophobe (KICH), kidney renal clear cell carcinoma (KIRC), kidney renal papillary cell carcinoma (KIRP), acute myeloid leukaemia (LAML), lower grade glioma (LGG), liver hepatocellular carcinoma (LIHC), lung adenocarcinoma (LUAD), lung squamous cell carcinoma (LUSC), mesothelioma (MESO), ovarian serous cystadenocarcinoma (OV), pancreatic adenocarcinoma (PAAD), pheochromocytoma and paraganglioma (PCPG), prostate adenocarcinoma (PRAD), rectum adenocarcinoma (READ), sarcoma (SARC), skin cutaneous melanoma (SKCM), stomach adenocarcinoma (STAD), testicular germ cell tumours (TGCT), thyroid carcinoma (THCA), thymoma (THYM), uterine corpus endometrial carcinoma (UCEC), uterine carcinosarcoma (UCS) and uveal melanoma (UVM)) types were available from The Cancer Genome Atlas (TCGA) database hosted at UCSC (https://xenabrowser.net/). We utilised Wilcoxon tests (log_2_(FPKM + 1)) to analyse the differential expression of LINC01929 across different cancer types. Subsequently, we analysed the expression of LINC01929 between paired samples. Moreover, we evaluated the expression of LINC01929 in patients at different stages of cancer.

### 2.2 Survival Analysis of LINC01929

We generated Kaplan–Meier survival curves to evaluate the prognostic significance of LINC01929 in terms of overall survival (OS), disease‐specific survival (DSS), and progression‐free interval (PFI) via the “survminer” and “survival” packages [19]. The cutoff value of high LINC01929 was defined as the top quartile, and the cutoff value of low LINC01929 was defined as the bottom quartile.

Correlations between LINC01929 and Tumour Mutational Burden, Microsatellite Instability, Immune Checkpoints and Infiltrating Immune Cells.

Tumours with high TMB often exhibit increased expression of neoantigens, while MSI‐H status may result in more mutations and the generation of additional neoantigens. These factors enhance the recognition and attack of tumours by the immune system, leading to a better response to immune checkpoint inhibitors and other immunotherapies. We investigated the relationship between LINC01929 expression and TMB/MSI via Spearman correlation analysis. Furthermore, the status of immune checkpoints has important implications for cancer treatment. We applied Spearman analysis to visualise the relationship between LINC01929 and immune checkpoints. The role of distinct immune cell types in modulating cancer progression, predicting prognosis and influencing therapy is increasingly emerging [20, 21]. We also calculated the proportion of infiltrating immune cells via single sample gene set enrichment analysis (ssGSEA) R scripts.

### 2.3 Cell Culture

We obtained SK‐BR‐3 and MDA‐MB‐231 cell lines from Procell Life Science & Technology Co. Ltd. (China). The cell lines were grown in DMEM (Gibco) supplemented with 10% FBS (Bioind) and 1% Pen‐Strep (Beyotime) at 37°C with 5% CO_2_.

### 2.4 Real‐Time PCR

We extracted RNA with TRIzol reagent (Invitrogen) and reverse transcribed to cDNA with Hiscript III RT SuperMix for qPCR (Vazyme). Real‐time PCR was performed with an Applied Biosystems system (Thermo Fisher Scientific) supplemented with SYBR Green (Vazyme). The sequences of primers used were as follows: β‐actin‐F: CATGTACGTTGCTATCCAGGC; β‐actin‐R: CTCCTTAATGTCACGCACGAT; LINC01929‐F:TCCTCTCATACCACTAACATC; LINC01929‐R: GCACCAACTTCAAGACAAT; TRAF2‐F: TCCCTGGAGTTGCTACAGC; TRAF2‐R: AGGCGGAGCACAGGTACTT; TNF‐α‐F: CCTCTCTCTAATCAGCCCTCTG; and TNF‐α‐R: GAGGACCTGGGAGTAGATGAG.

### 2.5 Knockdown of LINC01929

The cells were transfected with vectors, negative control (NC), or LINC01929 knockdown (SH) via Lipo8000 transfection reagent (Beyotime). Sh‐1: GGAAACCAGTAGGAGGCTATC, sh‐2: GACTTGACACGACTTCAGA. The lentiviral construct was optimised to achieve enhanced knockdown efficiency. Subsequently, the lentiviral transduction process was facilitated using polybrene as an assisting agent.

### 2.6 Transcriptome Sequencing Analysis Reveals the Correlation Between LINC01929 and Pathways

To investigate and validate the biological function of LINC01929, transcriptome sequencing analysis was conducted using a commercial service provider (Shanghai OE Biotech). Differential gene expression analysis was performed to identify genes whose expression significantly changed following LINC01929 knockdown. Gene Set Enrichment Analysis (GSEA) was also conducted to elucidate potential pathways associated with LINC01929.

### 2.7 Western Blotting

Cell lysis was performed for 40 min via RIPA lysis buffer. Subsequently, the samples were mixed with a loading buffer at 95°C for 10 min. The protein lysates were then separated via electrophoresis on a 10% SDS–PAGE gel system at a constant voltage of 120 V. Following electrophoresis, the proteins were transferred onto a PVDF membrane (Millipore). To prevent non‐specific binding, the membrane was blocked with bovine serum albumin for 2 h at room temperature. After overnight incubation at 4°C with the primary antibody, specific proteins were detected, followed by a 1‐h incubation at room temperature with the appropriate secondary antibody. Finally, the protein bands were visualised and analysed using ImageJ software for data quantification and analysis.

### 2.8 Cell Proliferation, Colony Formation and Cell Cycle Assays

We assessed cell proliferation with the CCK‐8 assay, seeding cells in 96‐well plates at a density of 4000 cells per well. Ten microlitres of CCK‐8 (Beyotime) reagent was added to each well, and the plates were incubated for approximately 1.5–2 h at 37°C. Cell viability was evaluated by comparing the absorbance (450 nm).

We used a colony formation assay to evaluate the long‐term growth potential of the cells. Cells were seeded in 6‐well plates at a density of 200–500 cells per well and cultured for 10–14 days until colonies became visible. The colonies were then fixed with paraformaldehyde and stained with crystal violet.

After 48–72 h of transfection, we collected and fixed cells in 75% ethanol. Then, we stained the cell with a propidium iodide solution containing RNase A (Beyotime) for 30 min at 37°C in the dark. The results were analysed with FlowJo_v10.8.1.

### 2.9 Xenograft Model

The animal experiments were carried out in compliance with the guidelines and regulations established by the ethical committee responsible for overseeing the welfare of the animals used in the research. To establish a subcutaneous xenograft tumour model using MDA‐MB‐231 cells, 6‐ to 8‐week‐old BALB/c‐nu mice were utilised. After 4 weeks, the mice were humanely euthanised, and the tumours were excised and measured to further investigate their characteristics. Tumour volume was calculated by measuring the length and width of the tumours with callipers.

### 2.10 Statistical Analysis

Wilcoxon tests were applied to determine the differential expression of LINC01929 between tumour and normal tissues. Kaplan–Meier curves were drawn to evaluate the prognostic relevance of LINC01929 with respect to OS, DSS and the PFI using Cox regression analysis, aiming to capture any statistically significant differences in survival outcomes. Spearman correlation analysis was applied to assess the potential correlation between LINC01929 expression and the TMB, MSI and immune checkpoint marker levels. The statistical analysis and visualisation described above were conducted using R 4.2.1, ImageJ and GraphPad Prism 9. Statistical significance was determined based on the following thresholds: *p* < 0.05 (*), *p* < 0.01 (**) and *p* < 0.001 (***).

## 3 Results

To elucidate the organisational framework of this manuscript, we have provided a delineation of the Workflow (Figure S1).

### 3.1 The Expression of LINC01929 in Pancancer

**FIGURE 1:**
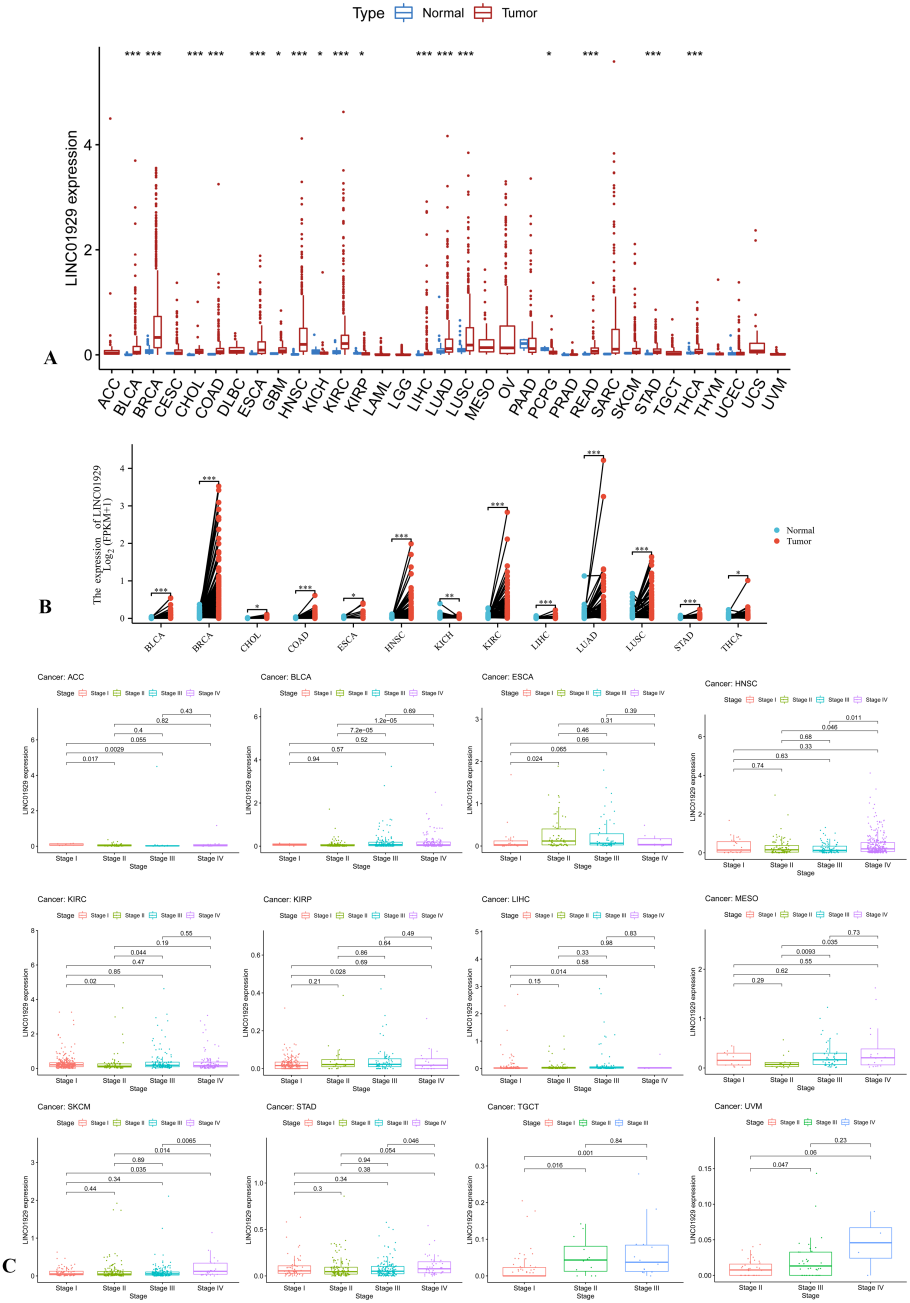
Expression of LINC01929 in various tumours and normal tissues. (A) Expression of LINC01929 in 33 different tumour and normal tissues. (B) Comparison of LINC01929 expression in paired samples from 13 types of tumours. (C) Results of the differential expression of LINC01929 in patients with different tumour stages. ACC, Adrenocortical carcinoma; BLCA, Bladder urothelial carcinoma; BRCA, Breast invasive carcinoma; CESC, Cervical squamous cell carcinoma and endocervical adenocarcinoma; CHOL, Cholangiocarcinoma; COAD, Colon adenocarcinoma; DLBC, Diffuse large B‐cell lymphoma; ESCA, Oesophageal carcinoma; GBM, Glioblastoma multiforme; HNSC, Head and neck squamous cell carcinoma; KICH, Kidney chromophobe; KIRC, Kidney renal clear cell carcinoma; KIRP, Kidney renal papillary cell carcinoma; LAML, Acute myeloid leukaemia; LGG, Lower grade glioma; LIHC, Liver hepatocellular carcinoma; LUAD, Lung adenocarcinoma; LUSC, Lung squamous cell carcinoma; MESO, Mesothelioma; OV, Ovarian serous cystadenocarcinoma; PAAD, Pancreatic adenocarcinoma; PCPG, Pheochromocytoma and paraganglioma; PRAD, Prostate adenocarcinoma; READ, Rectum adenocarcinoma; SARC, Sarcoma; SKCM, Skin cutaneous melanoma; STAD, Stomach adenocarcinoma; TGCT, Testicular germ cell tumours; THCA, Thyroid carcinoma; THYM, Thymoma; UCEC, Uterine corpus endometrial carcinoma; UCS, Uterine carcinosarcoma; UVM, Uveal melanoma. (*p < 0.05, **p < 0.01, ***p < 0.001).

The Wilcoxon test was used to compare the expression of LINC01929 between tumour and normal tissues among 33 tumour types. LINC01929 was highly expressed in 14 (BLCA, BRCA, CHOL, COAD, ESCA, GBM, HNSC, KIRC, LIHC, LUAD, LUSC, READ, STAD and THCA) common types of tumours and was expressed at low levels in three (KICH, KIRP and PCPG) types of tumours (Figure 1A). Moreover, in paired sample comparisons, LINC01929 exhibited increased expression in 12 (BLCA, BRCA, CHOL, COAD, ESCA, HNSC, KIRC, LIHC, LUAD, LUSC, STAD and THCA) types of tumours but decreased expression in KICH (Figure 1B). Furthermore, we observed distinct variations in LINC01929 expression across different stages within 12 tumour types (ACC, BLCA, ESCA, HNSC, KIRC, KIRP, LIHC, MESO, SKCM, STAD, TGCT and UVM) (Figure 1C).

### 3.2 Pancancer Prognostic Value of LINC01929

**FIGURE 2:**
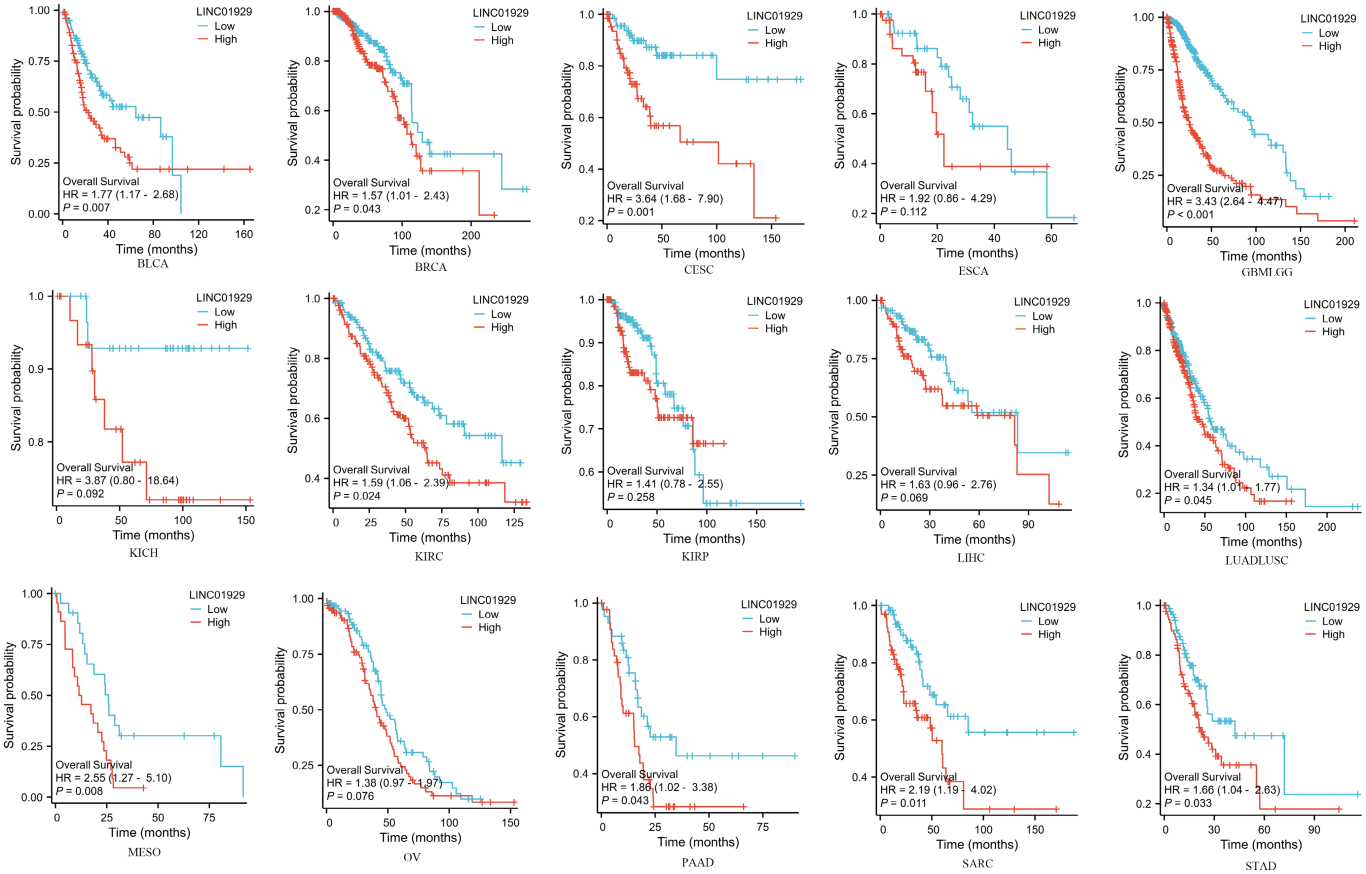
Impact of LINC01929 on overall survival in 15 types of tumours. BLCA, Bladder urothelial carcinoma; BRCA, Breast invasive carcinoma; CESC, Cervical squamous cell carcinoma and endocervical adenocarcinoma; ESCA, Oesophageal carcinoma; GBMLGG, Glioblastoma multiforme and lower grade glioma; HR, Hazard ratio; KICH, Kidney chromophobe; KIRC, Kidney renal clear cell carcinoma; KIRP, Kidney renal papillary cell carcinoma; LIHC, Liver hepatocellular carcinoma; LUADLUSC, Lung adenocarcinoma and lung squamous cell carcinoma; MESO, Mesothelioma; OV, Ovarian serous cystadenocarcinoma; PAAD, Pancreatic adenocarcinoma; SARC, Sarcoma; STAD, Stomach adenocarcinoma.

**FIGURE 3:**
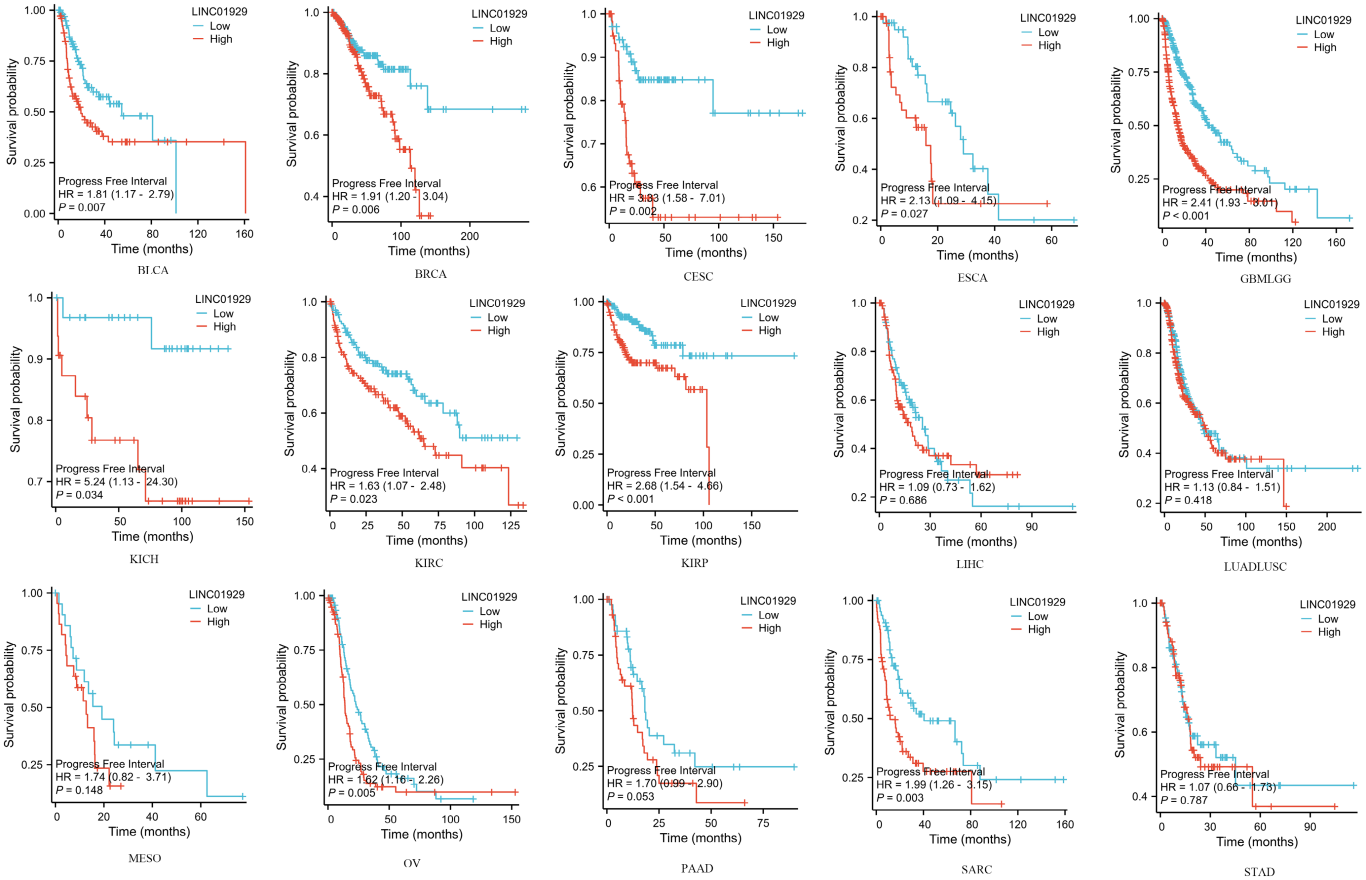
Impact of LINC01929 on the progression‐free survival interval in 15 tumour types. BLCA, Bladder urothelial carcinoma; BRCA, Breast invasive carcinoma; CESC, Cervical squamous cell carcinoma and endocervical adenocarcinoma; ESCA, Oesophageal carcinoma; GBMLGG, Glioblastoma multiforme and lower grade glioma; HR, Hazard ratio; KICH, Kidney chromophobe; KIRC, Kidney renal clear cell carcinoma; KIRP, Kidney renal papillary cell carcinoma; LIHC, Liver hepatocellular carcinoma; LUADLUSC, Lung adenocarcinoma and lung squamous cell carcinoma; MESO, Mesothelioma; OV, Ovarian serous cystadenocarcinoma; PAAD, Pancreatic adenocarcinoma; SARC, Sarcoma; STAD, Stomach adenocarcinoma.

**FIGURE 4:**
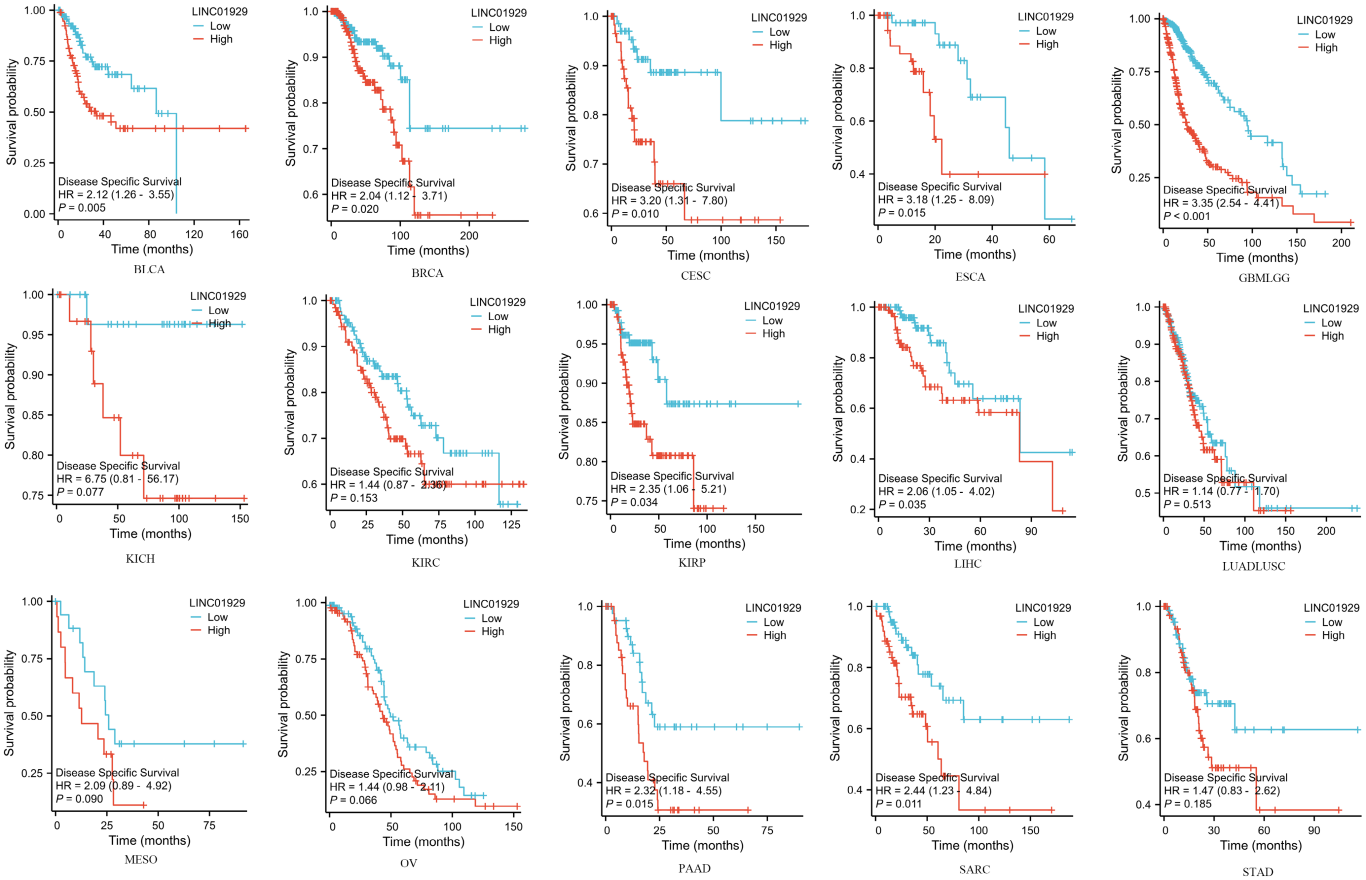
Impact of LINC01929 on disease‐specific survival in 15 types of tumours. BLCA, Bladder urothelial carcinoma; BRCA, Breast invasive carcinoma; CESC, Cervical squamous cell carcinoma and endocervical adenocarcinoma; ESCA, Oesophageal carcinoma; GBMLGG, Glioblastoma multiforme and lower grade glioma; HR, Hazard ratio; KICH, Kidney chromophobe; KIRC, Kidney renal clear cell carcinoma; KIRP, Kidney renal papillary cell carcinoma; LIHC, Liver hepatocellular carcinoma; LUADLUSC, Lung adenocarcinoma and lung squamous cell carcinoma; MESO, Mesothelioma; OV, Ovarian serous cystadenocarcinoma; PAAD, Pancreatic adenocarcinoma; SARC, Sarcoma; STAD, Stomach adenocarcinoma.

Kaplan–Meier plots of OS, DSS and PFI were drawn to evaluate the prognostic signature of LINC01929. Our findings indicated that LINC01929 acted as an oncogene and exerted a detrimental impact on prognosis (OS, DSS or PFI) in 15 distinct types of tumours. Our research revealed that LINC01929 expression was associated with unfavourable OS in patients with 10 types of tumours (Figure 2). Additionally, LINC01929 was associated with adverse PFI in 10 types of tumours (Figure 3) and poor DSS in 9 types of tumours (Figure 4). These results indicated that LINC01929 may act as an oncogenic role in a diverse array of tumour types.

### 3.3 Correlations Between LINC01929 and the TMB, MSI, Immune Checkpoint and Infiltrating Immune Cells

**FIGURE 5:**
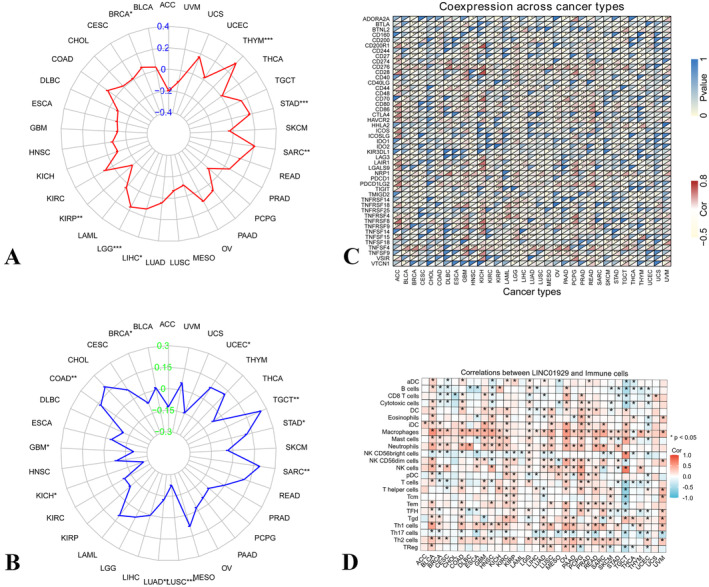
Relationships between LINC01929 expression and tumour mutational burden, microsatellite instability and immune checkpoint. (A) Association between LINC01929 expression and tumour mutational burden in 33 types of tumours. (B) Relationships between LINC01929 and microsatellite instability. (C) The correlation between LINC01929 and immune checkpoints. ACC, Adrenocortical carcinoma; BLCA, Bladder urothelial carcinoma; BRCA, Breast invasive carcinoma; CESC, Cervical squamous cell carcinoma and endocervical adenocarcinoma; CHOL, Cholangiocarcinoma; COAD, Colon adenocarcinoma; DLBC, Diffuse large B‐cell lymphoma; ESCA, Oesophageal carcinoma; GBM, Glioblastoma multiforme; HNSC, Head and neck squamous cell carcinoma; KICH, Kidney chromophobe; KIRC, Kidney renal clear cell carcinoma; KIRP, Kidney renal papillary cell carcinoma; LAML, Acute myeloid leukaemia; LGG, Lower grade glioma; LIHC, Liver hepatocellular carcinoma; LUAD, Lung adenocarcinoma; LUSC, Lung squamous cell carcinoma; MESO, Mesothelioma; OV, Ovarian serous cystadenocarcinoma; PAAD, Pancreatic adenocarcinoma; PCPG, Pheochromocytoma and paraganglioma; PRAD, Prostate adenocarcinoma; READ, Rectum adenocarcinoma; SARC, Sarcoma; SKCM, Skin cutaneous melanoma; STAD, Stomach adenocarcinoma; TGCT, Testicular germ cell tumours; THCA, Thyroid carcinoma; THYM, Thymoma; UCEC, Uterine corpus endometrial carcinoma; UCS, Uterine carcinosarcoma; UVM, Uveal melanoma. (*p < 0.05, **p < 0.01, ***p < 0.001).

In BRCA, THYM, STAD, SARC, LIHC and LGG, expression of LINC01929 was positively associated with TMB, while in KIRP, expression of LINC01929 was negatively associated with TMB (Figure 5A). Furthermore, LINC01929 expression exhibited a positive correlation with MSI in COAD, SARC, STAD, TGCT and UCSC and a negative correlation with MSI in BRCA, GBM, KICH, LUAD and LUSC (Figure 5B). Moreover, according to the heatmap of immune checkpoint genes, LINC01929 was shown to be associated with various immune checkpoint markers, such as NRP1 (neuropilin 1), CD276, PDCD1LG2 (programmed cell death protein 1 ligand 2), CD80, CD86 and TNF receptor‐related checkpoint proteins (Figure 5C). Immune checkpoints are closely related to immune cell infiltration. We found that LINC01929 is associated with the proportion of macrophages in various tumours and negatively correlated with CD8+ T cells. This may the reason for differences in patient prognosis (Figure 5D).

### 3.4 Downregulation of LINC01929 Inhibits Proliferation and Cell Cycle Progression in Breast Cancer Cells

The results of the expression analysis, prognostic analysis, TMB, MSI and immune checkpoint analysis revealed that LINC01929 was differentially expressed and had prognostic significance in breast cancer. To further validate these findings, appropriate breast cancer cell lines (SK‐BR‐3 and MDA‐MB‐231) were selected for in vitro and in vivo experiments.

**FIGURE 6:**
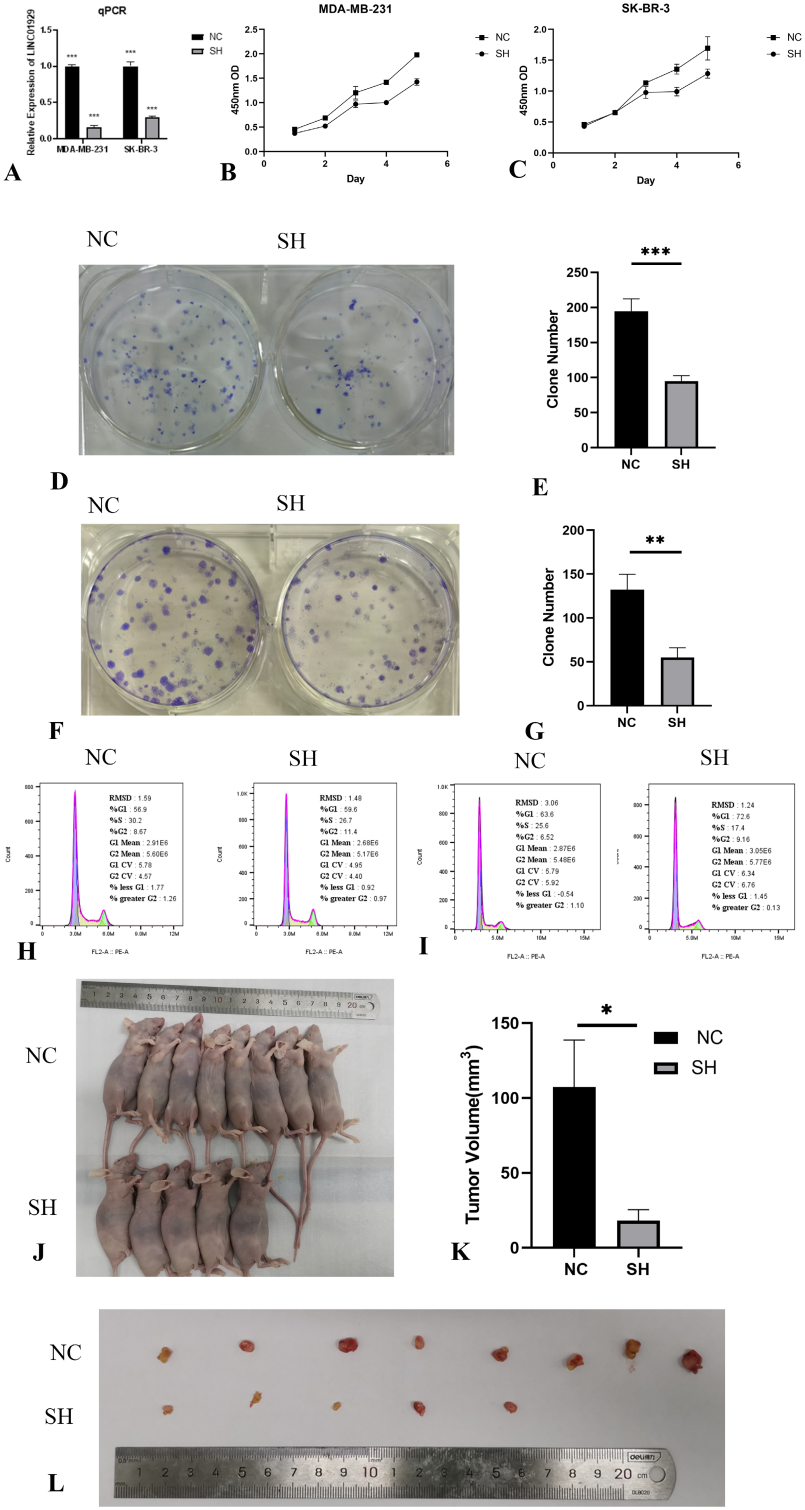
Suppression of LINC01929 inhibits cell proliferation and the cell cycle in breast cancer cell lines. (A) The expression of LINC01929 was downregulated in MDA‐MB‐231 and SK‐BR‐3 cells, as confirmed by qPCR. (B) Inhibition of cell viability in the MDA‐MB‐231 cell line upon LINC01929 knockdown. (C) Inhibition of cell viability in the SK‐BR‐3 cell line upon LINC01929 knockdown. (D) Suppression of colony formation in MDA‐MB‐231 cells following LINC01929 knockdown. (E) Statistical analysis of the colony formation assay results for MDA‐MB‐231 cells. (F) Suppression of colony formation in SK‐BR‐3 cells following LINC01929 knockdown. (G) Statistical analysis of the colony formation assay results for SK‐BR‐3 cells. (H) Cell cycle arrest in MDA‐MB‐231 cells upon LINC01929 knockdown. (I) Cell cycle arrest in SK‐BR‐3 cells upon LINC01929 knockdown. (J) Suppression of in vivo tumour growth upon LINC01929 knockdown. (K) Inhibition of tumour volume upon LINC01929 knockdown. (L) Status of tumours after LINC01929 knockdown. NC, Negative control; SH, LINC01929 knockdown. (*p < 0.05, **p < 0.01, ***p < 0.001).

The expression of LINC01929 was compared between NC and SH cells using qPCR analysis. LINC01929 was successfully knocked down in the SK‐BR‐3 and MDA‐MB‐231 cell lines (Figure 6A). Subsequent CCK8 assays suggested a significant decrease in cell proliferation following LINC01929 knockdown (Figure 6B,C). Moreover, plate colony formation assays revealed a notable decrease in the clonogenic potential of the cells (Figure 6D–G). Flow cytometry analysis further indicated cell cycle arrest after LINC01929 knockdown (Figure 6H,I). Notably, in the xenograft mouse model, out of the eight mice subjected to LINC01929 knockdown, three failed to develop tumours, while the remaining mice exhibited a substantial reduction in tumour volume (Figure 6J–L). These findings indicated the function of LINC01929 in cell proliferation and cell cycle progression.

### 3.5 Knockdown of LINC01929 Suppresses the TNF Signalling Pathway

**FIGURE 7:**
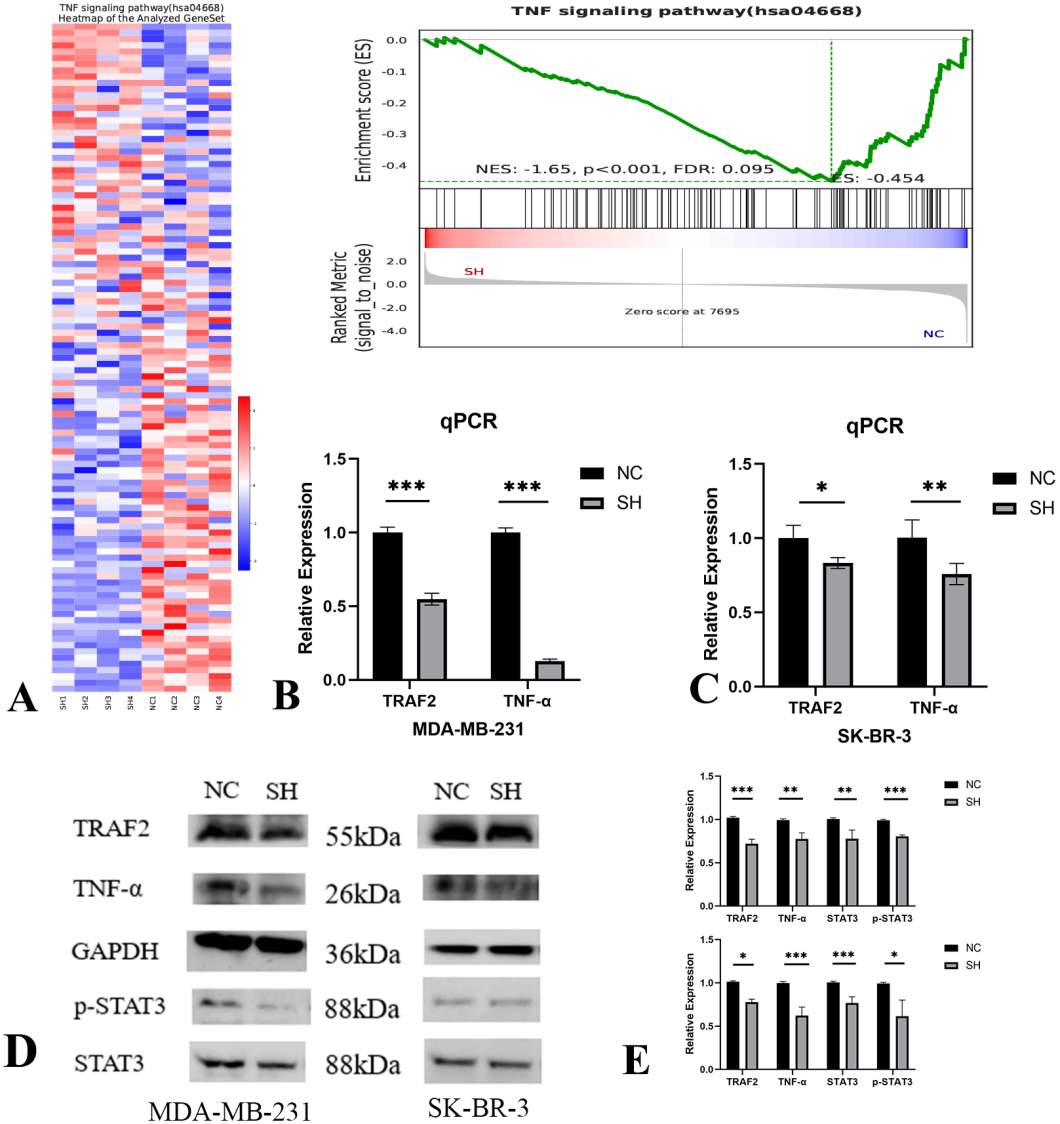
Suppression of LINC01929 inhibits the TNF pathway and STAT3. (A) Transcriptome sequencing results and a heatmap and GSEA plot demonstrating the inhibition of the TNF pathway upon LINC01929 knockdown. (B) Suppression of TRAF2 and TNF‐α mRNA expression upon LINC01929 knockdown in MDA‐MB‐231 cell lines. (C) Suppression of TRAF2 and TNF‐α mRNA expression upon LINC01929 knockdown in SK‐BR‐3 cell lines. (D) Inhibition of TRAF2, TNF‐α, and downstream STAT3 protein expression upon LINC01929 knockdown. (E) Statistical analysis of the western blot data. NC, Negative control; SH, LINC01929 knockdown. (*p < 0.05, **p < 0.01, ***p < 0.001).

To explore the molecular biological functions of LINC01929, RNA sequencing was performed to identify DEGs between NC and SH MDA‐MB‐231 cells. The heatmap and GSEA enrichment analysis suggested that knocking down LINC01929 inhibited the TNF signalling pathway (Figure 7A). Validation by RT–qPCR confirmed that knockdown of LINC01929 resulted in downregulation of TRAF2 and TNF‐α (biomarkers of the TNF signalling pathway; Figure 7B,C). Western blot analysis further demonstrated that downregulation of LINC01929 inhibited the expression of TRAF2, TNF‐α and downstream STAT3 (Figure 7D,E).

### 3.6 The Cytokine TNF‐α Activates the TNF Signalling Pathway and STAT3 to Promote Cell Cycle Progression

**FIGURE 8:**
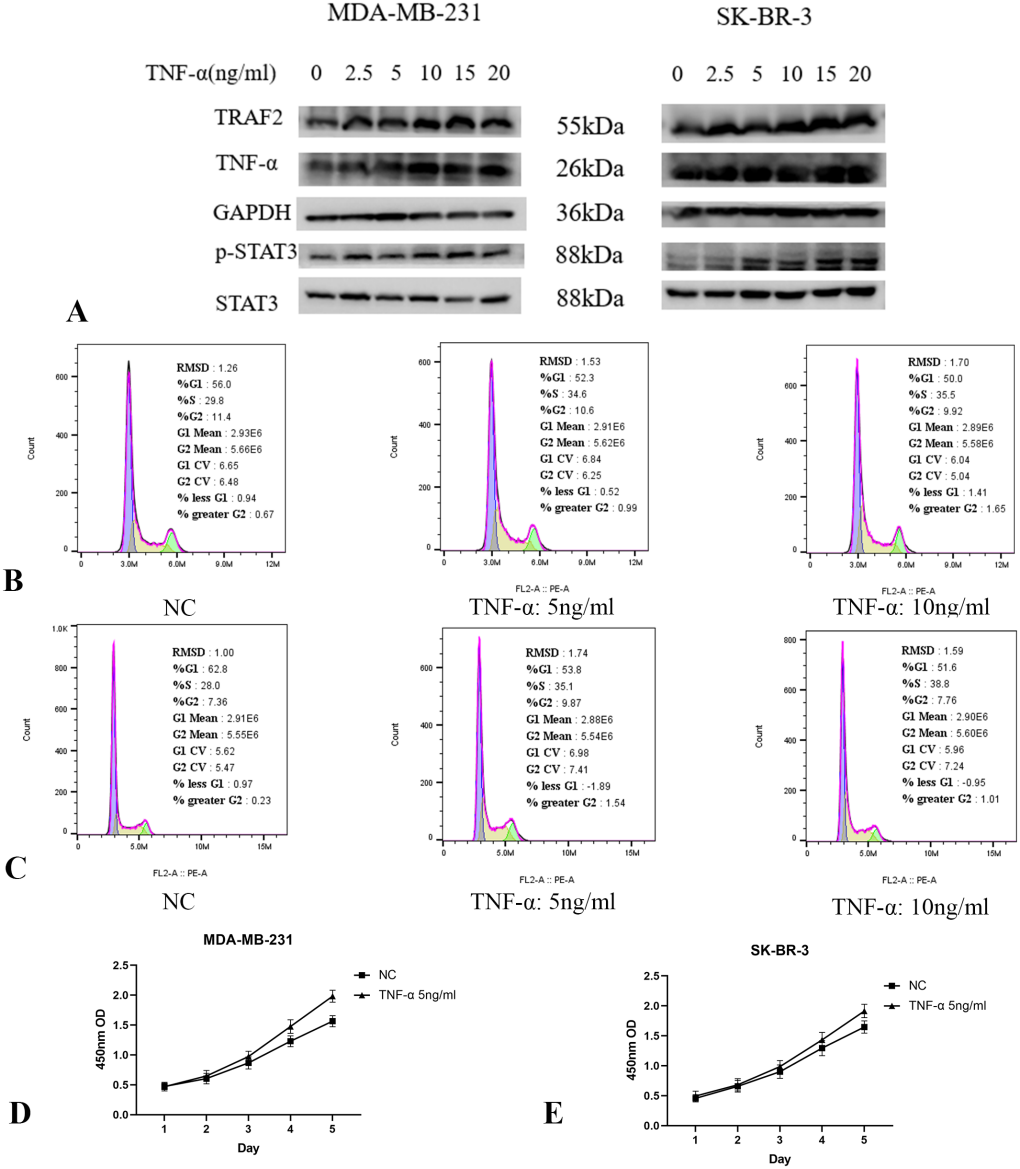
TNF‐α activates the TNF pathway and STAT3 and promotes cell cycle progression. (A) The cytokine TNF‐α activates the TNF pathway and downstream STAT3. (B) TNF‐α promotes cell cycle progression in MDA‐MB‐231 cells. (C) TNF‐α promotes cell cycle progression in SK‐BR‐3 cells. (D) TNF‐α promotes cell proliferation in MDA‐MB‐231 cells. (E) TNF‐α promotes cell proliferation in SK‐BR‐3 cells.

TNF‐α, a cytokine that binds to its receptor, activates the TNF pathway [22]. Additionally, STAT3, a transcription factor associated with cancer development, acts as a crucial mediator of TNF pathway activation [23]. To investigate whether the inhibition of STAT3 by LINC01929 knockdown is mediated by TNF, we discovered that the cytokine TNF‐α is capable of activating both the TNF pathway and STAT3 in breast cancer cell lines (Figure 8A). Additionally, our findings suggest that TNF‐α enhances cell cycle progression (Figure 8B,C) and cell proliferation (Figure 8D,E) in the MDA‐MB‐231 and SK‐BR‐3 cell lines.

### 3.7 LINC01929 Knockdown Suppresses Proliferation and Cell Cycle Progression via the TNF Signalling Pathway

**FIGURE 9:**
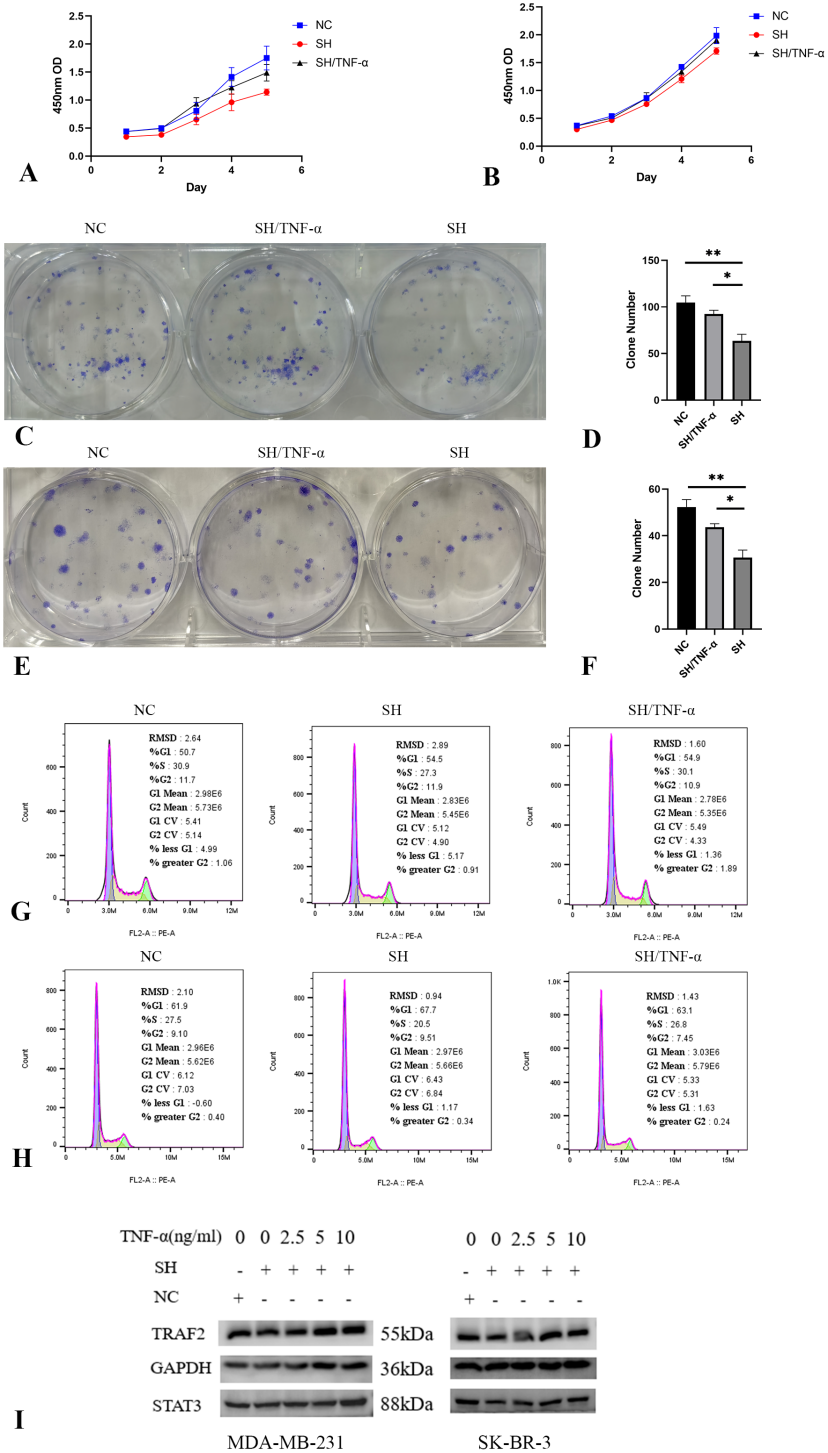
LINC01929 influences cell proliferation, the cell cycle, and STAT3 through the TNF pathway. (A) TNF‐α alleviates the cell proliferation inhibition caused by LINC01929 knockdown in MDA‐MB‐231 cells. (B) TNF‐α alleviates the cell proliferation inhibition caused by LINC01929 knockdown in SK‐BR‐3 cells. (C) TNF‐α alleviates the cell colony formation inhibition caused by LINC01929 knockdown in MDA‐MB‐231 cells. (D) Statistical results of cell cloning. (E) TNF‐α alleviates the cell colony formation inhibition caused by LINC01929 knockdown in SK‐BR‐3 cells. (F) Statistical analysis of the cell cloning results. (G) TNF‐α alleviates the cell cycle arrest caused by LINC01929 knockdown in MDA‐MB‐231 cells. (H) TNF‐α alleviates the cell cycle arrest caused by LINC01929 knockdown in SK‐BR‐3 cells. (I) TNF‐α alleviates the suppression of TRAF2 and STAT3 caused by LINC01929 knockdown in breast cancer cells. NC, Negative control; SH, LINC01929 knockdown. (*p < 0.05, **p < 0.01).

To validate the modulation of cell proliferation and cell cycle progression by LINC01929 through the TNF pathway, we performed LINC01929 knockdown and TNF‐α stimulation experiments in breast cancer cells. Our findings revealed that TNF‐α can effectively alleviate the changes in cell proliferation (Figure 9A–F) and cell cycle progression (Figure 9G,H) induced by LINC01929 knockdown. These results suggest that LINC01929‐induced effects on cell proliferation and the cell cycle are significantly relieved through activation of the TNF signalling pathway.

### 3.8 LINC01929 Knockdown Suppresses STAT3 by Inhibiting the TNF Signalling Pathway

Based on our previous findings, the downregulation of LINC01929 has been shown to inhibit the activation of STAT3 and the TNF pathway. Conversely, TNF‐α has been found to activate both the STAT3 pathway and the TNF pathway. Therefore, we hypothesised that the downregulation of LINC01929 modulates the expression of STAT3 via TNF pathway. To validate this hypothesis, we conducted experiments in which we knocked down LINC01929 and subsequently stimulated the cells with TNF‐α. Remarkably, our results demonstrated that the addition of exogenous TNF‐α effectively mitigated the downregulation of STAT3 induced by LINC01929 knockdown (Figure 9I). These findings provide compelling evidence supporting our hypothesis that the TNF pathway plays a crucial role in mediating the regulatory effects of LINC01929 on STAT3 in breast cancer cells. These findings may yield novel therapeutic strategies targeting the LINC01929/TNF/STAT3 axis in various diseases.

## 4 Discussion

Due to the rising incidence and mortality rates, cancer is regarded as a significant public health concern [24, 25]. Current therapeutic strategies, including surgery, radiotherapy, chemotherapy, and targeted therapies, have improved survival outcomes. However, various challenges, such as drug resistance and tumour recurrence continue to pose substantial barriers to effective treatment. Consequently, there is an urgent need for innovative biomarkers and therapeutic targets, which can enhance diagnostic and treatment paradigms for cancer patients. This research focuses on the expression and functional implications of LINC01929 in pan‐cancer, aiming to unravel its potential biological mechanisms and clinical significance. By employing a multifaceted approach, it was found that LINC01929 was highly expressed in various tumour types and was correlated with poor OS, DSS, and PFI in several cancers, including BRCA. We also Meanwhile, this study proved that the downregulation of LINC01929 significantly inhibited cancer cell growth and induced cell cycle arrest potentially through modulation of the TNF signalling pathway and its downstream STAT3. These insights not only advance the understanding of contributions of LINC01929 to cancer progression but also lay the groundwork for its potential application as a novel therapeutic target and prognostic biomarker in clinical settings.

The expression profile of LINC01929 across various cancer types underscores its potential as an oncogenic biomarker, with notably high expression in multiple malignancies, including breast cancer, and this high expression suggests its pivotal role in tumour biology. Kaplan–Meier survival analyses further reveal the prognostic significance of LINC01929, which highlights its relevance in clinical oncology. The correlation between LINC01929 expression and poor overall survival in diverse cancer types suggests that it could serve as a valuable prognostic marker for patients' stratification and management. In vitro experiments demonstrate that LINC01929 promotes breast cancer cell proliferation and survival, with significant growth inhibition observed following LINC01929 knockdown, which emphasises its critical function in cell cycle regulation and paves the way for its potential use as a prognostic indicator or therapeutic target in personalised cancer treatment strategies.

Subsequently, the relationship between LINC01929 and immune response parameters was explored, which offered further insights into its role in cancer progression. Our results indicate that LINC01929 expression is correlated with TMB and MSI in specific cancer types, which suggests its involvement in the immune landscape of tumours [26, 27, 28]. Moreover, the analysis of immune checkpoint genes suggested a compelling link between LINC01929 expression and TNF‐related immune checkpoint expression across multiple tumour types, which implicates its role in modulating inflammatory responses within the tumour microenvironment [29, 30]. Through immune infiltration analysis, it was found that the expression of LINC01929 is positively correlated with macrophages and negatively correlated with CD8 T cells in various tumours, which suggested that it may act as a modulator of immune evasion, a hallmark of cancer [31, 32, 33, 34]. These findings provide a rationale for investigating LINC01929 as a target to enhance the efficacy of immunotherapies. A deeper understanding of how LINC01929 influences the immune response could inform the development of combination strategies, which could leverage its expression to improve patient outcomes in immunotherapy.

In vivo and in vitro experiments suggested that LINC01929 plays a key regulator in tumour growth. The knockdown of LINC01929 significantly inhibited breast cancer cell proliferation and induced cell cycle arrest. These findings are echoed in the literature, where LINC01929 has been implicated in the regulation of proliferation, migration and invasion in various cancer types, which emphasises the potential of targeting LINC01929 for therapeutic intervention [15, 17, 18].

The mechanistic insights provided by RNA sequencing indicate that LINC01929 may exert its effects through the modulation of the TNF/ STAT3 signalling pathway. TNF/STAT3 pathway participates in the regulation of diverse biological processes and ultimately plays a crucial role in tumour formation, metastasis, and drug resistance. A study that examined 93 breast cancer specimens revealed a positive correlation between TNF‐α expression on tumour cell surfaces and lymph node metastasis [35]. Furthermore, a comparative analysis of TNF‐α expression in the serum of breast cancer patients and healthy individuals revealed significantly greater TNF‐α expression in breast cancer patients. Additionally, elevated TNF‐α levels were observed in patients with larger tumours (> 5 cm), greater lymph node metastasis, and higher TNM stage than in early‐stage patients [36]. The activation of the TNF signalling pathway can induce the activation of STAT3, thus resulting in its dimerisation, translocation into the nucleus, and subsequent binding to the promoter regions of target genes [37, 38, 39, 40]. Understanding the specific interactions between LINC01929 and the TNF pathway may unveil new therapeutic avenues targeting inflammatory and immune pathways in cancer management. Future investigations should delineate the broader signalling networks influenced by LINC01929, aiming to establish its role not only as an oncogenic factor but also as a crucial player in tumour‐host interactions that influence cancer progression and therapeutic responses.

According to this study, it was observed that the LINC01929 gene, an important pan‐cancer biomarker, exhibits high expression across various types of cancer and is associated with poor prognosis. This finding supports the important role of LINC01929 in tumorigenesis and provides a potential basis for early diagnosis and prognostic management. However, reliance on single‐gene detection methods has certain limitations in clinical applications. Given the complexity and heterogeneity of cancer, relying solely on LINC01929 as a single‐gene marker may not comprehensively capture the biological characteristics of tumours. Research indicates that interactions among multiple genes have significant impacts on cancer development and prognosis. Using multi‐gene combinations for comprehensive analysis can provide a more thorough assessment of cancer risk and prognosis [41, 42, 43, 44]. In recent years, machine learning techniques have offered new perspectives for multi‐gene analysis. Therefore, by constructing machine learning models that incorporate the expression levels of multiple genes, researchers can identify complex gene interaction networks, thereby improving the accuracy of cancer classification. Based on tumour‐associated non‐coding RNAs, machine learning prediction models may represent more promising approaches in the future. While providing significant insights into the expression and potential roles of LINC01929 across various cancer types, the reliance on bioinformatics analyses inherently lacks the validation that comes from direct clinical sample assessments. Furthermore, the sample sizes utilised in the analyses may not fully encompass the heterogeneity present within the broader cancer population, thereby potentially limiting the generalizability of the results. Future research should integrate in vitro and in vivo studies with clinical data to substantiate these findings and elucidate the clinical implications of LINC01929 in cancer biology.

In conclusion, this research underscores the potential of LINC01929 as a pivotal player in cancer biology, particularly in breast cancer. The observed correlations between LINC01929 expression and various prognostic factors highlight its prospective utility as a biomarker for patient stratification and therapeutic intervention. By delineating the role of LINC01929 in tumour progression and its relationship with key signalling pathways, this study lays the groundwork for future investigation of developing targeted therapeutic strategies. Most importantly, the implications of these findings extend beyond breast cancer, which suggests that LINC01929 may be a viable target for intervention across multiple malignancies, thereby contributing to the advancement of personalised medicine in oncology.

## Author Contributions

**Yanlin Gu:** software (lead), validation (equal), writing – original draft (equal). **Zhengyang Feng:** methodology (lead), supervision (equal). **Xiaoyan Xu:** conceptualization (lead), data curation (lead), resources (lead). **Liyan Jin:** formal analysis (lead), validation (equal), writing – original draft (equal). **Guoqin Jiang:** funding acquisition (lead), supervision (equal), writing – review and editing (lead).

## Ethics Statement

This study was conducted in accordance with the principles outlined in the Declaration of Helsinki. The Ethics Committee of The Second Affiliated Hospital of Soochow University granted approval for this research (Number: JD‐BS‐2022‐0033).

## Conflicts of Interest

The authors declare no conflicts of interest.

## Supporting information


Figure S1

## Data Availability

The data analysed in this study could be obtained from TCGA database.
