# Supplementary figures and images for: LINC01929 Is a Prognostic Biomarker for Multiple Tumours and Promotes Cell Proliferation in Breast Cancer Through the TNF/STAT3 Axis
Source: J Cell Mol Med. 2024 Nov 25;28(22):e70227. doi: 10.1111/jcmm.70227 (PMC11588430; doi:10.1111/jcmm.70227)

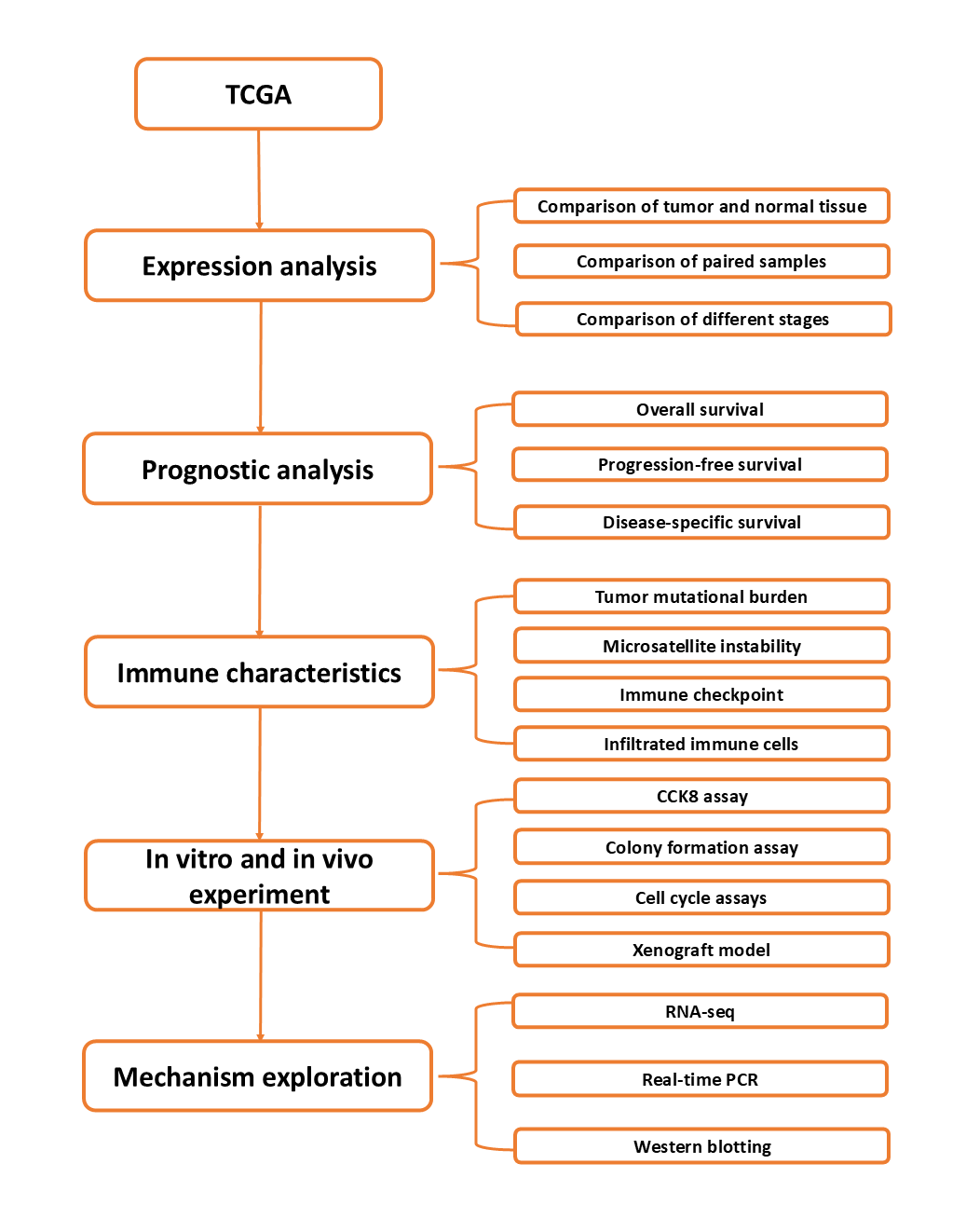

Supplement: Supplementary file 1 — Figure S1 [file JCMM-28-e70227-s001.tif]
